# Exploring the Epidemiological Characteristics and Survival Analysis Among Prostate Cancer Patients Under 50: A Seer‐Based Population Study

**DOI:** 10.1002/hsr2.70414

**Published:** 2025-01-24

**Authors:** Bahaa Mali, Ali Mali, Alaa Mali, Mohammed Abdulrazzak, Afnan W. M. Jobran

**Affiliations:** ^1^ Faculty of Medicine Al Quds University Jerusalem Palestine; ^2^ Faculty of Medicine University of Aleppo Aleppo Syria

**Keywords:** incidence rates, prostate cancer, SEER, survival rates, young men

## Abstract

**Background and Aims:**

Even though aging is a known risk factor for prostate cancer incidence and mortality, there has been an increase in incidence among young men since the late 1980s with notably lower survival rates than those among older men. However, there is insufficient knowledge about recent trends in the incidence and survival of this disease.

**Methods:**

We analyzed prostatic cancer incidence trends in men under 50 from 1975 to 2020 using Surveillance, Epidemiology, and End Results (SEER) 8 registries data. We further studied frequency, incidence rate, and survival rates in this group across SEER 22 registries, which cover around 41.9% of the US population. We analyzed the data by age, stage, and race subgroups and identified potential risk factors affecting survival prognosis using multivariable Cox proportional hazards regression models.

**Results:**

Our results revealed that prostate cancer incidence rates in men under 50 have increased from 1975 to 2020. There was a significant decline from 2009 to 2014, followed by a gradual decrease. Between 2004 and 2020, 35,670 new cases were diagnosed. 76.10% of these cases were localized, 15.10% were regional, 4.20% were distant, and 4.60% were unstaged. Certain factors can negatively impact the prognosis, including age at diagnosis under 30, advanced stages of the disease, and non‐Hispanic black race.

**Conclusion:**

Early‐onset prostate cancer has distinct epidemiological and clinical characteristics, and more research is required to gain a better understanding of the biological, genetic, and environmental factors that contribute to its development. This understanding will assist in the creation of more suitable management plans that can enhance survival rates.

**Abbreviations**
CI: confidence interval
NHAIAN: non‐Hispanic American Indian/Alaska Native
NHAPI: non‐Hispanic Asian or Pacific Islander
NHB: non‐Hispanic Black
NHUR: non‐Hispanic unknown race
NHW: non‐Hispanic White

## 1 Introduction

Prostate cancer is a common cancer in males globally, with approximately 1.6 million cases and 366,000 deaths reported every year [1]. In most cases, prostate cancers are identified in asymptomatic patients through a screening study that shows an elevated level of prostate‐specific antigen (PSA) or through findings on a digital rectal examination (DRE).

Aging is a well‐known risk factor for prostate cancer incidence and mortality globally, with the average age at diagnosis being 66 years [2]. However, early observation revealed a rise in incidence in the 20–49‐year and 50–55‐year age groups since the late 1980s and early 1990s [3]. A report by Bleyer et al. showed that Survival rates were worse in the United States among older adolescents and young adults than older men, with 5‐year survival rates of 30%–50% in those diagnosed under the age of 30 years [4]. These observations suggest that early‐onset prostate cancer has a unique biological etiology and clinical sequelae that require further investigation.

There is insufficient knowledge about the recent trend in this disease incidence and survival, particularly with the recent decline in incidence in the older age groups [5]. In this study, we aimed to investigate the trend in prostatic cancer incidence in under 50 years age men from 1975 to 2020 using surveillance, epidemiology, and end results (SEER) 8 registries data [6]. Additionally, we studied the epidemiological and survival characteristics of the disease in this specific young age group by analyzing (SEER Research Limited, 22 registries), which covers approximately 41.9% of the US population [7].

## 2 Methods

### 2.1 Study Cohort Selection

We defined our target patient population as under‐50‐year‐old men with prostatic carcinoma using (prostate site) according to the International Classification of Diseases for Oncology, Third Edition, code C619, and with malignant behavior.

### 2.2 Trend Analysis

We collected data on prostate cancer incidence between 1975 and 2020 from SEER 8 registries, which cover about 8.3% of the US population. Incidence rates were calculated per 100,000 men and age‐adjusted to the 2000 US standard population. Confidence intervals were 95% for rates (Tiwari method). We utilized Joinpoint Regression software to calculate the average percent change (APC) and identify trends in incidence rates. The software can detect the years when the APC trend shifts upward or downward, and it determines whether these trends are statistically significant.

### 2.3 Frequency and Incidence Rate by Age, Stage, and Race

We analyzed the frequency and incidence rate using SEER 22 registries, which offer the largest SEER geographic coverage available. For subgroup analysis, we categorized our analysis based on stage, and race. Stage stratification was done using the Combined Summary Stage (2004+) and was divided into localized, regional, distant, and unknown/Unstaged subgroups. Similarly, race stratification was done using non‐Hispanic White, non‐Hispanic Black, Non‐Hispanic American Indian/Alaska Native, non‐Hispanic Asian or Pacific Islander, Hispanic (all races), and Non‐Hispanic Unknown Race categories. Our analysis was limited to patients diagnosed from 2004 to 2020 due to changes in stage coding. We calculated incidence rates per 100,000 men and age‐adjusted them to the 2000 US standard population. The confidence intervals for rates were set at 95%.

### 2.4 Ethics Statement

This study was based on anonymized data from a publicly available dataset; therefore, ethical approval was not required. Moreover, the data used in this research has been obtained from a publicly available dataset and does not contain any personally identifiable information.

### 2.5 Survival Analysis and Multivariable Cox Proportional Hazards Regression

Using 2004–2015 data from SEER 22 registries, we calculated one‐ and 5‐year relative cancer survival rates. The SEER defines relative survival as a measure of net survival that represents cancer survival in the absence of other causes of death. Relative survival was calculated as the ratio of observed to expected survival while adjusting for the general survival rate of the US population for that race, sex, age, and date at which the age was coded. We used log‐rank analysis to compare overall survival differences between different age, race, and stage subgroups. Additionally, we used multivariable Cox proportional hazards regression models to identify potential risk factors for survival prognosis. We processed and analyzed the data using R statistical software Version 4.3.2, and statistical significance was defined as *p* < 0.05.

## 3 Results

### 3.1 Incidence Trends (1975–2020)

**Figure 1:**
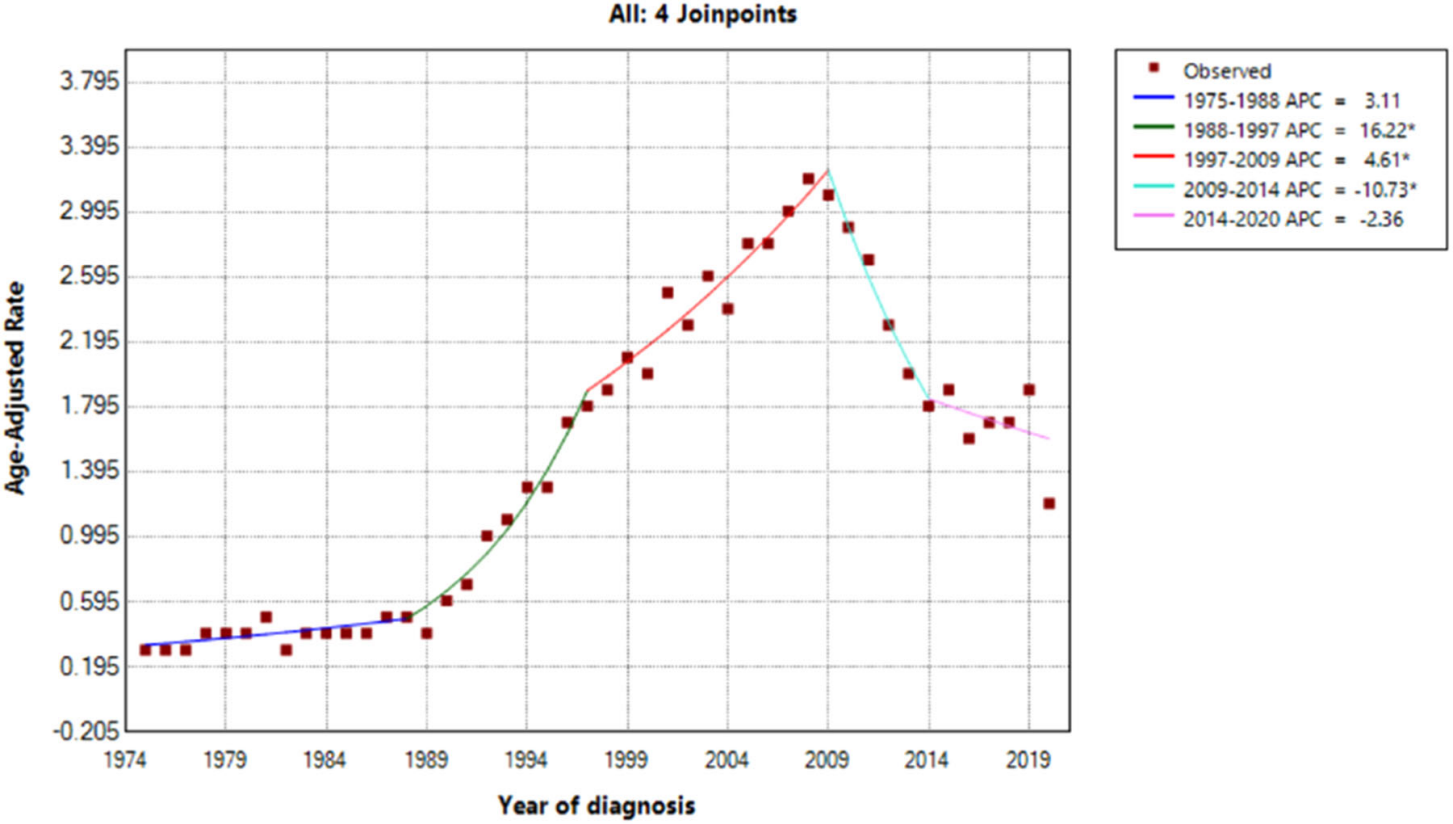
Prostatic cancer age‐adjusted incidence trends in under‐50‐year‐old men based on SEER 8 (1975–2020).

The overall age‐adjusted incidence rate of prostate cancer in men under 50 years old has increased from 1975 to 2020 with an average annual percent change (AAPC) of 3.58, which is statistically significant (*p* < 0.05). The sharpest rise was observed since 1998, with an annual percent change of 16.22. However, the incidence rate of the disease declined sharply from 2009 to 2014 with an APC of −10.73 (*p* < 0.05), and then declined more gradually from 2014 to 2020 (Figure 1).

### 3.2 Frequency and Incidence Rate by Stage, and Race (2004–2020)

Between 2004 and 2020, there were 35,670 newly diagnosed cases of prostate cancer in men under 50 years of age. The age‐adjusted incidence rate was 2.4 per 100,000. Of these cases, 76.10% were localized, 15.10% were regional, 4.20% were distant, and 4.60% were unstaged. Non‐Hispanic white men accounted for 54.70% of cases, followed by non‐Hispanic black men at 27.90%, Hispanic men at 12.80%, non‐Hispanic Asian or Pacific Islander men at 2.40%, non‐Hispanic men of unknown race at 1.80%, and non‐Hispanic American Indian/Alaska Native at 0.30%. The age‐adjusted incidence rate was highest among non‐Hispanic black men at 5.60, followed by non‐Hispanic white men at 2.4 (Table 1).

**Table 1:** Counts and incidence rates for prostate cancer by race and stage in < 50‐year‐old US men from 2004 to 2020, based on SEER Research Limited field data, 22 registries.

Variable	Count	(*n*, %)	Rate*	95% CI
Total	35,670	100.00%	2.4	2.4–2.4
Race				
NHW	19,510	54.70% (19,510/35,670)	2.4	2.4–2.4
NHB	9966	27.90% (9966/35,670)	5.6	5.5–5.8
NHAIAN	115	0.30% (115/35,670)	1.1	0.9–1.4
NHAPI	863	2.40% (863/35,670)	0.6	0.6–0.7
Hispanic	4564	12.80% (4564/35,670)	1.3	1.3–1.4
NHUR	652	1.80% (652/35,670)	—	—
Stage				
Localized	27,160	76.10% (27,160/35,670)	1.8	1.8–1.8
Regional	5391	15.10% (5391/35,670)	0.4	0.4–0.4
Distant	1491	4.20% (1491/35,670)	0.1	0.1–0.1
Unstaged	1628	4.60% (1628/35,670)	0.1	0.1–0.1

* Rates are per 100,000 men and are age‐adjusted to the 2000 US standard population.

### 3.3 Survival Analysis and Multivariable Cox Proportional Hazards Regression

The 5‐year relative survival rate for men diagnosed with prostate cancer in the United States is generally favorable, with a rate of 100% for those diagnosed with local, 98.4% for regional stage and 93.6% for unknown/unstaged cancer. However, for those diagnosed with distant‐stage cancer, the rate drops significantly to 32.00%. Men diagnosed with prostate cancer under the age of 50 have an overall 5‐year relative survival rate of 97.40%, while those under the age of 30 have a rate of 59.30%. In terms of race, the 5‐year relative survival rates are 97.80% for non‐Hispanic White, 97.60% for non‐Hispanic Black, 93.30% for non‐Hispanic American Indian/Alaska Native, 97.80% for non‐Hispanic Asian or Pacific Islander, 94.60% for Hispanic and 99.70% for non‐Hispanic Unknown Race.

**Figure 2:**
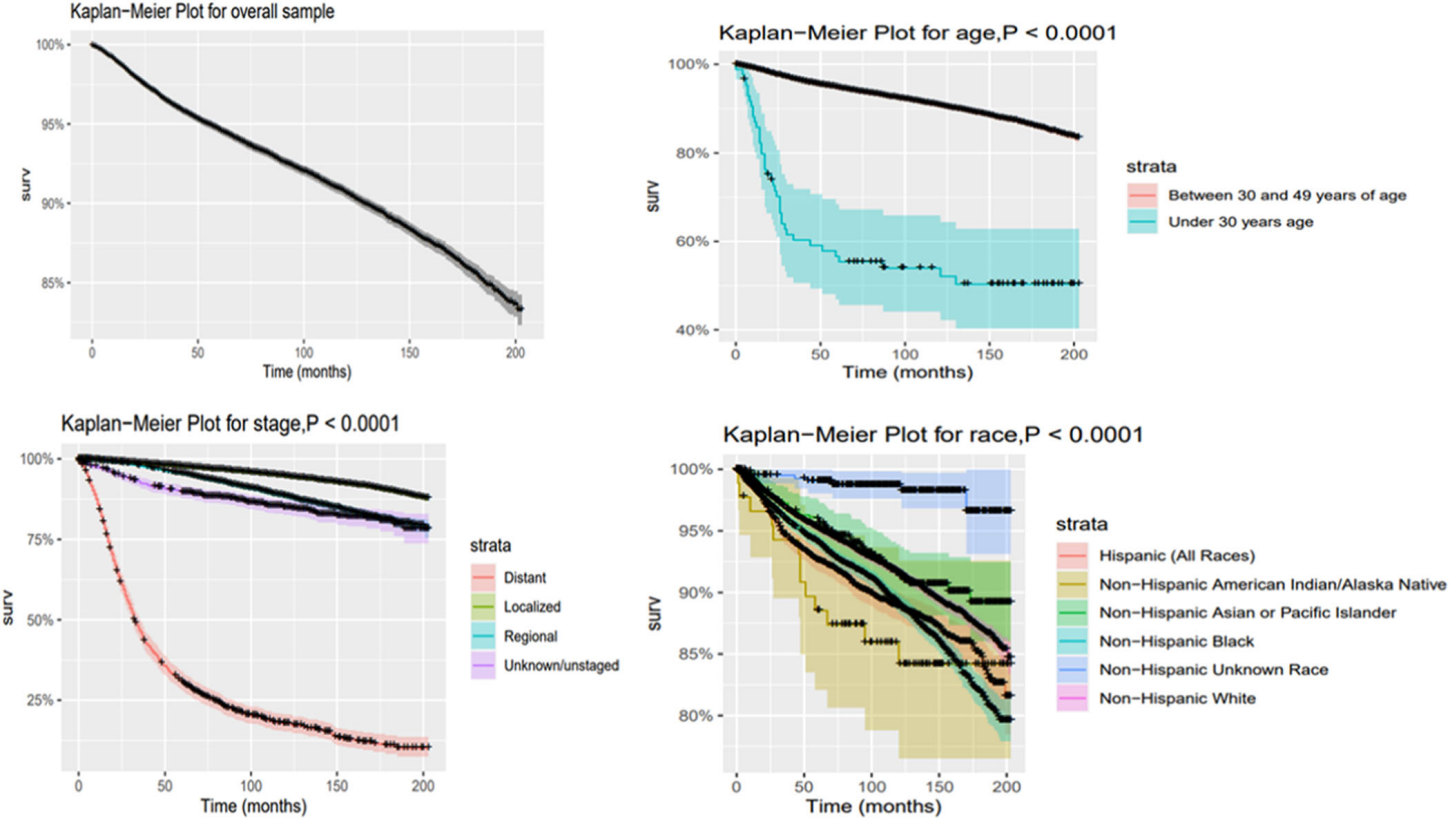
Kaplan‐Meier curves show overall survival rates for men under 50 diagnosed with prostate cancer in the US from 2004 to 2015. The data is categorized by age, race, and stage and collected from SEER Research field data in 22 registries.

Overall survival rates based on Kaplan‐Meier estimates vary depending on age, race, and stage factors (Table 2; Figure 2). Patients under the age of 30 have lower survival rates after 1 and 5 years of diagnosis compared to those in the 30‐ to 49‐year‐old age group (*p* < 0.0001, log‐rank test). The summary stages also have a significant impact on survival, with the distant stage showing the lowest one‐ and 5‐year survival rates at 84% and 30.8%, respectively. However, the localized stage has the highest overall survival rates after 1 and 5 years of diagnosis, at 99.5% and 97.6%, respectively. In terms of race, non‐Hispanic black, non‐Hispanic American Indian/Alaska Native, and Hispanic races have lower one‐ and 5‐year survival rates than non‐Hispanic white races. However, higher survival rates are noticed in non‐Hispanic, unknown races. The results of the univariable Cox models are consistent with these findings, except that the non‐Hispanic American Indian/Alaska Native race did not have a statistically significant impact on survival. Additionally, the multivariable analysis showed that the Hispanic race did not have a statistically significant impact on survival (Table 3).

**Table 2:** Survival rates based on Kaplan‐Meier analysis; sub grouped by different variables.

Variable	Survival % (95% CI) at 1 year	Survival % (95% CI) at 5 years
Total	98.9 (98.8–99.0)	94.6 (94.4–94.9)
Age		
30–49	98.9 (98.8–99.0)	94.7 (94.5–95.0)
< 30	85.8 (78.7–93.6)	56.6 (46.9–68.4)
Race		
NHW	99.1 (99.0–99.3)	95.2 (94.9–95.5)
NHB	98.5 (98.2–98.8)	94.1 (93.5–94.6)
NHAIAN	96.6 (92.8–100.0)	88.5 (82.1–95.5)
NHAPI	99.4 (98.7–100.0)	95.9 (94.4–97.5)
Hispanic	98.6 (98.2–99.0)	92.6 (91.7–93.5)
NHUR	99.5 (98.8–100.0)	99.0 (98.0–100.0)
Stage		
Localized	99.5 (99.4–99.6)	97.6 (97.4–97.8)
Regional	99.5 (99.2–99.7)	95.5 (94.9–96.1)
Distant	84.0 (81.8–86.4)	30.8 (28.0–33.8)
Unstaged	97.3 (96.4–98.3)	89.7 (87.8–91.6)

**Table 3:** Univariate and multivariate cox regression models for the following factors: age at diagnosis, race and summary stages.

		Univariate regression		Multivariate regression	
		HR (95% CI)	*p* value	HR (95% CI)	*p* value
Age (vs. 30–49)	< 30	6.47 (4.74–8.84)	*p* < 0.001	1.56 (1.14–2.14)	*p* = 0.006
Race (vs. NHW)	Hispanic	1.32 (1.18–1.47)	*p* < 0.001	1.08 (0.96–1.20)	*p* = 0.192
	NHAIAN	1.60 (0.93–2.77)	*p* = 0.090	—	—
	NHAPI	0.90 (0.68–1.18)	*p* = 0.433	—	—
	NHB	1.36 (1.25–1.47)	*p* < 0.001	1.30 (1.20–1.41)	*p* < 0.001
	NHUR	0.19 (0.09–0.39)	*p* < 0.001	0.21 (0.10–0.45)	*p* < 0.001
Stage (vs. Localized)	Distant	35.01 (32.00–38.30)	*p* < 0.001	34.03 (31.07–37.28)	*p* < 0.001
	Regional	2.10 (1.91–2.32)	*p* < 0.001	2.10 (1.90–2.31)	*p* < 0.001
	Unstaged	2.72 (2.31–3.21)	*p* < 0.001	2.79 (2.37–3.30)	*p* < 0.001

## 4 Discussion

Our analysis of the trend in age‐adjusted incidence rates of prostate cancer in men under the age of 50 has shown an overall rise in incidence rates from 1975 to 2020. The AAPC during this period was 3.58%. This increase is consistent with earlier observations of the rise in age‐adjusted incidence since the late 1980s and early 1990s [3, 4]. However, we also noticed a sharp decline in incidence rates from 2009 to 2014, with an APC of −10.73% (*p* < 0.05). This decline was followed by a more gradual decline from 2014 to 2020. Our observations of this sharp decline after 2009 in younger age groups are similar to those reported previously in older age groups [5, 8, 9].

Based on SEER data from the years 2000 to 2020, we found that the majority of prostate cancer cases diagnosed in younger than 50‐year‐old men were localized, with a percentage of 76.10%. 15.10% of cases were regional, 4.20% were distant, and 4.60% were unstaged. Other authors report 1,310,373 newly diagnosed cases of prostate cancer in the US between 2001 and 2007. They found that 81.6% of cases were localized, 10.0% were regional, 3.1% were distant, and 5.3% were unstaged [10]. These findings suggest that even though the majority of diagnosed cases in patients under 50 years old were localized, there is a higher likelihood of being diagnosed with a more advanced disease compared to patients over 50 years old. This can be explained by the fact that prostate cancer is generally considered a disease of older people, which delays its consideration and diagnosis in younger age groups.

Non‐Hispanic black patients exhibited a higher incidence of prostate cancer and a more severe prognosis. Statistical significance was found in both univariate and multivariate Cox regression analyses. The racial disparities observed in our study, particularly the higher incidence and worse prognosis in non‐Hispanic Black men, may be driven by a combination of genetic, socioeconomic, and environmental factors. Previous research has identified a genetic predisposition in men of African descent, including faster progression from latent to aggressive prostate cancer [11]. Additionally, socioeconomic factors such as limited access to healthcare, lower rates of insurance coverage, and delayed diagnosis in minority groups may exacerbate these disparities. Other authors report that African‐American and Afro‐Caribbean men show comparable tendencies after radical prostatectomy, with more severe disease features and a poorer 5‐year biochemical recurrence‐free survival rate than Caucasian‐American males [12]. These findings imply a genetic contribution, particularly given the result of the study among men of West African descent in the Caribbean and South America, which found comparable incidence and mortality rates to black men in the United States [13]. However, these discrepancies are most likely attributable to a combination of genetic, environmental, and socioeconomic factors [14]. A more equitable healthcare system that provides early screening and treatment, regardless of socioeconomic status, could help mitigate these disparities. Future studies should further explore the interplay of genetic and social determinants of health to inform targeted interventions for high‐risk groups.

Our analysis has found that certain factors can negatively impact the prognosis of prostate cancer for patients under the age of 50. These factors include more advanced stages of the disease and being diagnosed at a younger age. Our study revealed that patients under the age of 30 had lower relative and overall survival rates compared to those between the ages of 30 and 49. This finding was statistically significant, with a higher hazard ratio in both univariate and multivariate analyses of 6.47 (*p* < 0.001) and 1.56 (*p* = 0.006), respectively. It is worth noting that several studies have also observed lower survival rates for the youngest patients, which were worse than for all other age groups except for men diagnosed at over 80 years old. Our findings of increasing incidence rates in men under 50, particularly the sharp rise from 1998 to 2009, have practical implications for clinical practice and healthcare policy. The early detection of prostate cancer in younger men, historically considered a disease of older populations, warrants a reevaluation of current screening guidelines. Earlier screening in high‐risk populations, such as men with a family history of prostate cancer or those of African descent, may be crucial [4, 15, 16].

Contrary to this, a US population‐based study found that the annual mortality rate for prostate cancer tends to increase with age at diagnosis and attained age. The study followed a group of patients who were diagnosed between 1992 and 1997 for 25 years. The study revealed that of the men who were diagnosed before the age of 60, 14.4% died from prostate cancer, while of those diagnosed after 70, 21.1% died from the disease. The exact reason behind the higher death rate in older men in the cohort of this study is not yet clear, but some factors like weakened immunity, comorbidity, and a lower likelihood of receiving curative treatment could contribute to it. However, the study only followed patients diagnosed with prostate cancer between 1992 and 1997, a period of significant increase in prostate cancer incidence due to PSA testing. Additionally, treatment of prostate cancer has changed significantly since 1997. These limitations may restrict generalization of the study's findings beyond this period and population [17].

Finally, we should shade a light on the limitations that warrant consideration. The retrospective nature of the study may introduce selection bias, as we relied on existing records without randomization, which could affect the generalizability of the results. Additionally, the Joinpoint regression model, while useful in detecting changes in trend, is sensitive to the number and positioning of the join points, potentially influencing the observed trends. We also did not account for treatment modalities, comorbidities, or socioeconomic factors, which are known to impact both survival and prognosis. These unmeasured variables may explain some of the differences in survival rates, particularly in younger patients, and should be investigated in future prospective studies. However, the large population in our cohort comes from the SEER database, which is a high‐quality registry with a rigorous quality assurance program. This clinical database captures a large population of cancer patients in the United States, making our findings generalizable.

## 5 Conclusion

Even though the earlier observation of an increase in the incidence of prostate cancer in younger age populations was true, there was a sharp decline in incidence rates from 2009 to 2014. This decline was followed by a more gradual decline from 2014 to 2020, which is consistent with what has been reported previously in older age groups. When it comes to being diagnosed with prostate cancer, there is a higher likelihood of being diagnosed with a more advanced disease in comparison to patients over 50 years old. Some factors can negatively impact the prognosis of prostate cancer in patients who are under the age of 50. These factors include being diagnosed before the age of 30, having a more advanced stage of the disease, and being non‐Hispanic black. It is crucial to further study the biological, genetic, and environmental differences of these cancers in younger populations as compared to older men. This understanding will help develop more appropriate management plans that can improve survival rates.

## Author Contributions

**Bahaa Mali:** conceptualization, validation, resources, project administration, writing–original draft, writing–review and editing. **Ali Mali:** conceptualization, methodology, validation, writing–review and editing; writing–original draft, formal analysis, resources. **Alaa Mali:** resources, writing–review and editing, writing–original draft, conceptualization, methodology, validation. **Mohammed Abdulrazzak:** writing–review and editing, software, resources. **Afnan W. M. Jobran:** conceptualization, resources, writing–review and editing, supervision.

## Ethics Statement

This study was based on anonymized data from a publicly available dataset; therefore, ethical approval was not required.

## Conflicts of Interest

The authors declare no conflicts of interest. All authors have read and approved the final version of the manuscript. Dr. Afnan W. M. Jobran had full access to all of the data in this study and takes complete responsibility for the integrity of the data and the accuracy of the data analysis.

## Transparency Statement

The lead author Dr. Bahaa Mali affirms that this manuscript is an honest, accurate, and transparent account of the study being reported; that no important aspects of the study have been omitted; and that any discrepancies from the study as planned (and, if relevant, registered) have been explained.

## Data Availability

The data that support the findings of this study are available from the corresponding author upon reasonable request.
